# An Unusual Case of Tetanus Masquerading as an Acute Abdomen: A Case Report

**DOI:** 10.5811/cpcem.2020.8.49073

**Published:** 2020-10-19

**Authors:** Rahul Kumar Thakur, Rajshree Singh, Sabin Nepal, Prasanna Ghimire

**Affiliations:** * B.P. Koirala Institute of Health Sciences, Department of Emergency Medicine, Dharan, Nepal; † Nepalese Army Institute of Health Sciences College of Medicine, Department of Emergency Medicine, Kathmandu, Nepal; ‡ Nepalgunj Medical College and Teaching Hospital, Department of Radiology, Bheri, Nepal

**Keywords:** Tetanus, acute abdomen, abdominal muscle spasm, toxoid, vaccine

## Abstract

**Introduction:**

Tetanus is an acute onset neurological disease that is often lethal. It has a high disease burden in low and middle-income countries. Tetanus is caused by a toxin made by spores of the bacterium *Clostridium tetani*, which are found in soil, dust, and animal feces. The toxin impairs the motor neurons leading to muscle stiffness. However, with the development of a toxoid vaccine, the incidence has sharply declined and is now categorized as a vaccine-preventable disease. The treatment of tetanus is primarily supportive and focuses on managing the complications until the effects of toxins resolve.

**Case Report:**

We report the case of a 67-year-old farmer who previously sustained a laceration injury approximately 45 days prior to presenting to the emergency department with abdominal pain and rigidity. After a comprehensive evaluation to rule out other items in the differential diagnoses, he was diagnosed with tetanus based on clinical symptoms and ultimately required mechanical ventilation. The patient was then managed in the intensive care unit and later made an uneventful recovery.

**Conclusion:**

This case illustrates an uncommon presentation of tetanus and the latency of the infectious process. Often when patients present with atypical symptoms, it poses a diagnostic dilemma to the clinicians. Thus, it is very important to carefully elicit a history of contaminated injury. This case also highlights the importance of prophylactic vaccine in low and middle-income countries, which can reduce disease-related mortality and morbidity.

## INTRODUCTION

Tetanus is an acute onset neurological disease caused by the bacterium *Clostridium tetani* and is often lethal. Causing an estimated 213,000–293,000 deaths worldwide annually, tetanus is a global health burden that disproportionately impacts developing countries.^1^ In 2016, the United States mortality rate was as low as 11% compared to 20% in developing countries.^2^ Although maternal and neonatal tetanus in low and middle-income countries (LMIC) is on the verge of elimination, there were significant cases of adult tetanus reported in the same year.^3^

Tetanus usually occurs in a wound contaminated with soil, manure, or rusted metal.^4^ In up to 50% of cases, the injury may be minor and is not considered significant enough to pursue medical treatment; and in15–25% of patients, there is no evidence of a recent wound at all.^4^ The incubation period averages 7–10 days, with a range of 1–60 days.^4^ The initial signs are trismus and risus sardonicus. As the disease progresses, patients may exhibit stiffness of the neck, difficulty in swallowing, opisthotonos, and rigidity of the pectoral, abdominal, and extremity muscle groups.^2^ Most patients achieve a complete but slow recovery with good functional outcomes; however, elderly patients are particularly at risk of functional impairment.^5^

## CASE REPORT

A 67-year-old farmer presented to the emergency department (ED) with severe abdominal pain, which had started as a mild, colicky pain in the left lower quadrant. Over a seven-day period, the pain intensified becoming more diffuse and associated with intermittent subjective fevers that prompted the patient to seek medical attention. He denied nausea, vomiting, gastrointestinal bleeding, constipation, and obstipation. He had no significant chronic medical or surgical history. The physical examination was significant for a rigid, diffusely tender abdomen, a normal digital rectal examination, and normal bowel sounds. The patient was afebrile and in distress with a normal oxygen saturation. His vital signs included a blood pressure of 140/90 millimeters of mercury, heart rate of 84 beats per minute, and a normal respiratory rate. Laboratory analysis showed normal complete blood counts, serum electrolytes and urinalysis, serum amylase and lipase. Electrocardiogram and chest radiograph were normal. Ultrasound of the abdomen was ordered. The patient was admitted with a provisional diagnosis of acute peritonitis.

Abdominal ultrasound was normal without evidence of intraperitoneal fluid collection or omental thickening. Subsequently, computed tomography (CT) of the abdomen and pelvis with intravenous (IV) contrast was performed after the patient’s symptoms failed to improve. The CT was significant for bilateral nephrolithiasis but without findings suggestive of peritonitis including ascites and mesenteric/omental fat- stranding or thickening.^6^ The patient was started on conservative treatment with IV fluids, analgesic, and broad-spectrum antimicrobial agents.

Over the next 24 hours, the patient developed opisthotonos confirming the clinical diagnosis of tetanus. Further directed history obtained from the patient’s wife revealed that he had sustained a contaminated laceration injury to his head approximately 45 days prior to his presentation to the ED. The wound was managed at a local medical store without any post-exposure, prophylaxis tetanus vaccine. Careful examination of the patient’s scalp revealed a healing wound. Shortly thereafter, he developed laryngeal spasm requiring mechanical ventilation and was transferred to the intensive care unit. His treatment included human tetanus immunoglobulins, broad-spectrum antibiotics, muscle relaxants comprising diazepam and baclofen, IV hydration, and parenteral nutrition. The patient was successfully weaned off the ventilator after a two-week course. He was then transferred to a general ward and discharged from hospital one week later. He did not suffer any major sequelae after being discharged.

CPC-EM Capsule

What do we already know about this clinical entity?

*Tetanus is caused by spores of* Clostridium tetani, *causing impairment of motor neurons, particularly affecting people in low to middle-income countries (LMIC)*.

What makes this presentation of disease reportable?

*We describe a rare case of tetanus presenting as an acute abdomen that could have led to misdiagnosis if pertinent history had been overlooked*.

What is the major learning point?

*In LMIC, patients presenting as acute abdomen and with a history of contaminated wounds should prompt the clinician to consider tetanus as a differential*.

How might this improve emergency medicine practice?

*This case reinforces the importance of prophylactic tetanus toxoid vaccine. especially in the elderly*.

## DISCUSSION

As demonstrated in this case, tetanus presenting as acute abdominal pain presents many challenges because it is uncommon, misleading, and non-specific to tetanus. Generalized rigidity with trismus is the most commonly reported symptom at onset in approximately 75% of cases.^7^ While uncommon, tetanus presenting as an acute abdomen in the ED has been reported by various authors over the last century.^7^–^10^

A patient presenting with an acute abdomen is frequently encountered in the ED and typical non-intraperitoneal differential diagnoses include muscle strain, rectus sheath hematoma, and herpes zoster. This case provides evidence that tetanus should also be included in the differential diagnosis of acute abdomen originating from the abdominal wall in patients from LMIC. Our patient’s epidemiological profile fits into this description with a relatively high likelihood of acquiring a tetanus infection. Risk factors include male gender, >65 years of age, farmer profession, late presentation without signs of wound infection, and lack of post-exposure, prophylaxis tetanus toxoid vaccine.^11^

Abdominal rigidity has also been demonstrated as a first sign suggestive of tetanus in older children and adults.^2^ The board-like abdominal rigidity is attributed to the spasm of abdominal muscles.^12^ Abdominal rigidity can also be a symptom of localized tetanus when the abdominal wall is involved as the site of injury. In an emergency setting, it is challenging to discern abdominal pain from rigidity (which is specific to neuromuscular diseases such as tetanus) and pain from intraperitoneal processes. After imaging rules out serious causes of acute abdomen such as perforated viscus, acute intestinal obstruction, aortic dissection, and mesenteric ischemia, a further history of traumatic injuries, wounds, or infection should be considered in a patient presenting with abdominal rigidity. Furthermore, it is difficult to distinguish abdominal rigidity from abdominal guarding (involuntary tightening secondary to pain), which can be overcome by having the patient purposely relax the muscles.^13^

Most cases of tetanus occur in unvaccinated individuals and adults.^2^ Studies show that patients over the age of 60 with inadequate immunity have antibodies below the 49–66% necessary protective level.^4^ In addition to providing post-exposure, tetanus toxoid vaccination in the emergency care setting to patients with open wounds and no vaccination in the prior 10 years, we recommend a booster dose every 10 years. The full course of vaccination regimen provides immunity to tetanus for 10 years in 95% cases.^14^ The immunization programs of many developing nations do not yet have a catch-up or booster immunization program targeted to the elderly. Providers practicing in such settings should not miss the opportunity to vaccinate people aged 65 years or older with combined tetanus, diphtheria and acellular pertussis vaccine (Tdap) in a non-emergency setting.^15^ We recommend booster immunization with Tdap because it is immunogenic in population 65 years or older, and has a good safety profile.

## CONCLUSION

Tetanus is a vaccine-preventable disease that is still prevalent in many low and middle-income countries, although it is rare in high-income countries. The differential diagnosis in non-immunized older children and adults with abdominal rigidity should include generalized tetanus. Awareness and high index of suspicion in developing countries are crucial for early diagnosis and prompt initiation of appropriate treatment to avoid unnecessary intervention and procedures.
